# Encapsulated Enigma: Surgical Resolution of a Lingual Lipoma Affecting Oral Functions

**DOI:** 10.1002/ccr3.71579

**Published:** 2026-02-15

**Authors:** Gabriele Delia, Fabiana Battaglia, Maria Lentini, Mariausilia Franchina, Ludovica Pepe, Francesco Stagno d’Alcontres

**Affiliations:** ^1^ Department of Plastic and Reconstructive Surgery University Hospital of Messina “AOU Gaetano Martino” Messina Italy; ^2^ Department of Human Pathology in Adult and Developmental Age “Gaetano Barresi” University of Messina Messina Italy

**Keywords:** adipose tumor, lingual lipoma, oral neoplasm, submucosal lesion, surgical excision

## Abstract

Lingual lipomas are uncommon benign tumors, accounting for < 0.5% of all tongue neoplasms, due to the limited adipose tissue in this region. They are usually asymptomatic and slow growing, which may delay detection. We report the case of a 50‐year‐old man with a 4‐year history of a painless, progressively enlarging submucosal mass on the ventral surface of the tongue. Clinical examination revealed a 3 × 3 cm sessile, yellowish, well‐circumscribed lesion with tense‐elastic consistency. Given its benign appearance, no preoperative imaging was performed. The lesion was surgically excised under local anesthesia, and histopathological analysis confirmed a conventional lipoma composed of mature adipocytes without atypia or lipoblasts. The patient recovered uneventfully and regained full oral function within 2 months. This case emphasizes the importance of including lingual lipomas in the differential diagnosis of tongue masses. It suggests that, in selected cases, clinical assessment alone may be sufficient for diagnosis and treatment planning.

## Key Clinical Message

Lingual lipomas are extremely rare due to the paucity of adipose tissue in the tongue, yet they should be considered in the differential diagnosis of submucosal oral lesions. Careful clinical evaluation may guide management, while surgical excision ensures definitive treatment and rapid functional recovery.

## 1 Introduction

Lipomas are the most common benign mesenchymal tumors and account for approximately 13%–20% of all soft tissue neoplasms. In the oral cavity, they represent about 1%–5% of all benign oral tumors [1, 2]. Despite their prevalence in the head and neck region, lingual lipomas are extremely rare, making up only about 0.3% of all tongue neoplasms [2]. This rarity is likely due to the limited amount of adipose tissue in the tongue, which restricts the development of lipomatous growths in this location.

Clinically, tongue lipomas present as slow‐growing, painless, well‐circumscribed, yellowish submucosal lesions, often discovered incidentally or when they reach a size that interferes with speech, mastication, or swallowing [2]. Functional impairment in such cases may significantly affect the patient's quality of life by interfering with essential oral functions, thus highlighting the clinical relevance of timely diagnosis and surgical management. Their consistency may range from soft to tense‐elastic, depending on the degree of encapsulation and the extent of surrounding tissue involvement. Histologically, several variants of lipoma have been described, including conventional lipoma, fibrolipoma, angiolipoma, spindle cell lipoma, and intramuscular (infiltrating) lipoma. The intramuscular subtype is relevant in the tongue because it may infiltrate muscle fibers, raising differential diagnostic concerns with liposarcoma [3, 4]. Although benign, lingual lipomas require histopathological confirmation to rule out malignancy, especially in the presence of atypical features or accelerated growth. This case report aimed to present the surgical management of a rare lingual lipoma with functional impairment to contribute to the improvement of the diagnosis process and treatment of such lesions.

## 2 Case History

A 50‐year‐old male patient presented with a slowly enlarging submucosal mass on the ventral surface of the anterior two‐thirds of the tongue, present for approximately 4 years. The lesion had progressively increased in size, becoming mildly bothersome due to recent interference with speech and mastication, though it remained completely painless.

**FIGURE 1:**
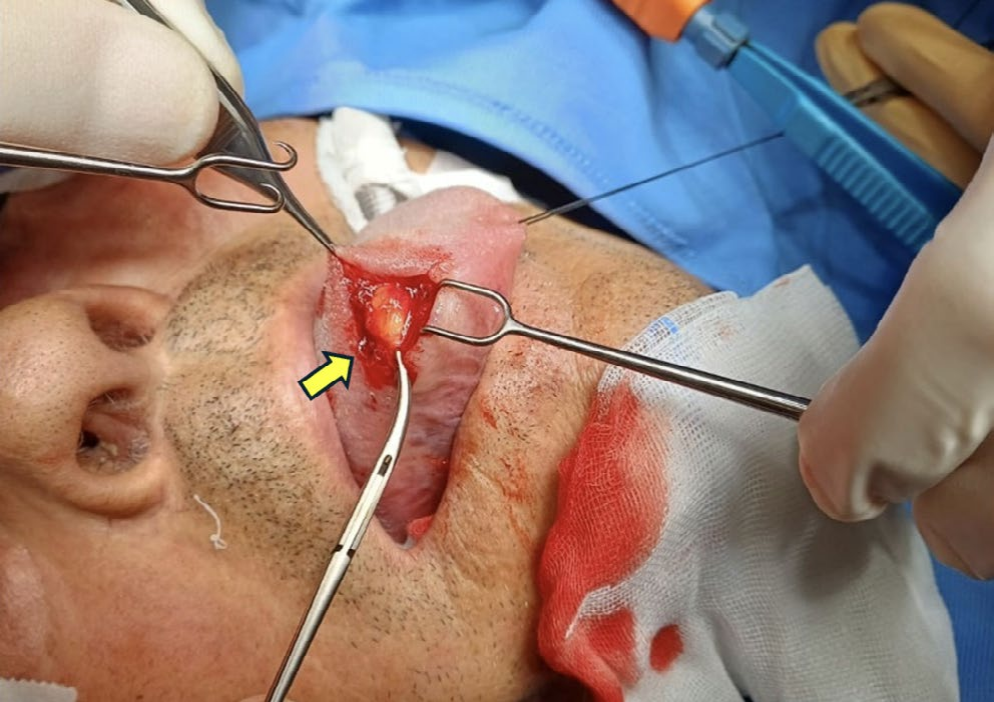
Intraoperative exposure of the yellowish submucosal mass on the ventral surface of the tongue (arrow indicating the lesion).

On clinical examination, a sessile, yellowish mass was observed on the left side of the tongue, measuring approximately 3 × 3 cm. The lesion was well‐circumscribed, with regular margins and a tense‐elastic consistency (Figure 1). The lesion exhibited a positive slip sign, indicating its mobility relative to the underlying structures and suggesting a well‐circumscribed, benign nature. No signs of ulceration, inflammation, or cervical lymphadenopathy were present. The patient had no relevant systemic illnesses or syndromic traits.

## 3 Differential Diagnosis, Investigations, and Treatment

Based on the clinical presentation, a benign soft tissue neoplasm was suspected. The differential diagnosis included fibrolipoma, granular cell tumor, and minor salivary gland adenoma. Given the typical clinical features of a lipoma: soft, mobile, well‐circumscribed, and noninfiltrative—imaging was deemed unnecessary.

**FIGURE 2:**
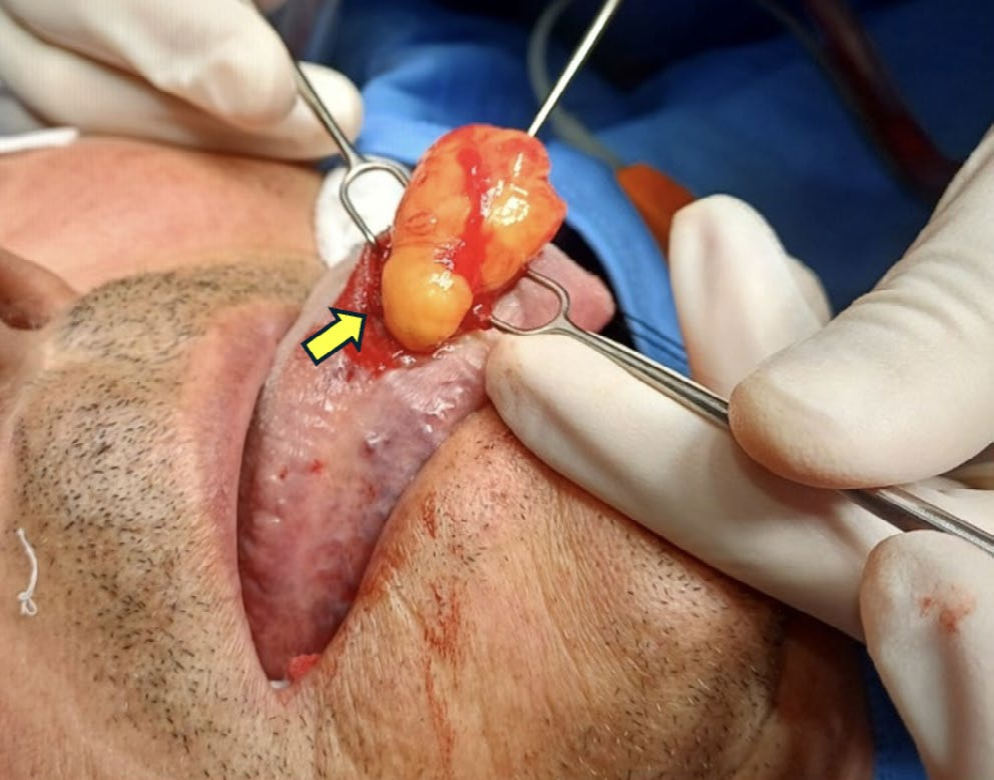
Intraoperative image showing progressive dissection and mobilization of the encapsulated lipomatous lesion (arrow indicating the lesion).

The lesion was surgically excised in its entirety under local anesthesia using an intraoral approach (Figure 2). Imaging was considered unnecessary as the lesion exhibited typical features of a benign lipoma: soft, mobile, well‐circumscribed, and without signs of infiltration or rapid growth.

**FIGURE 3:**
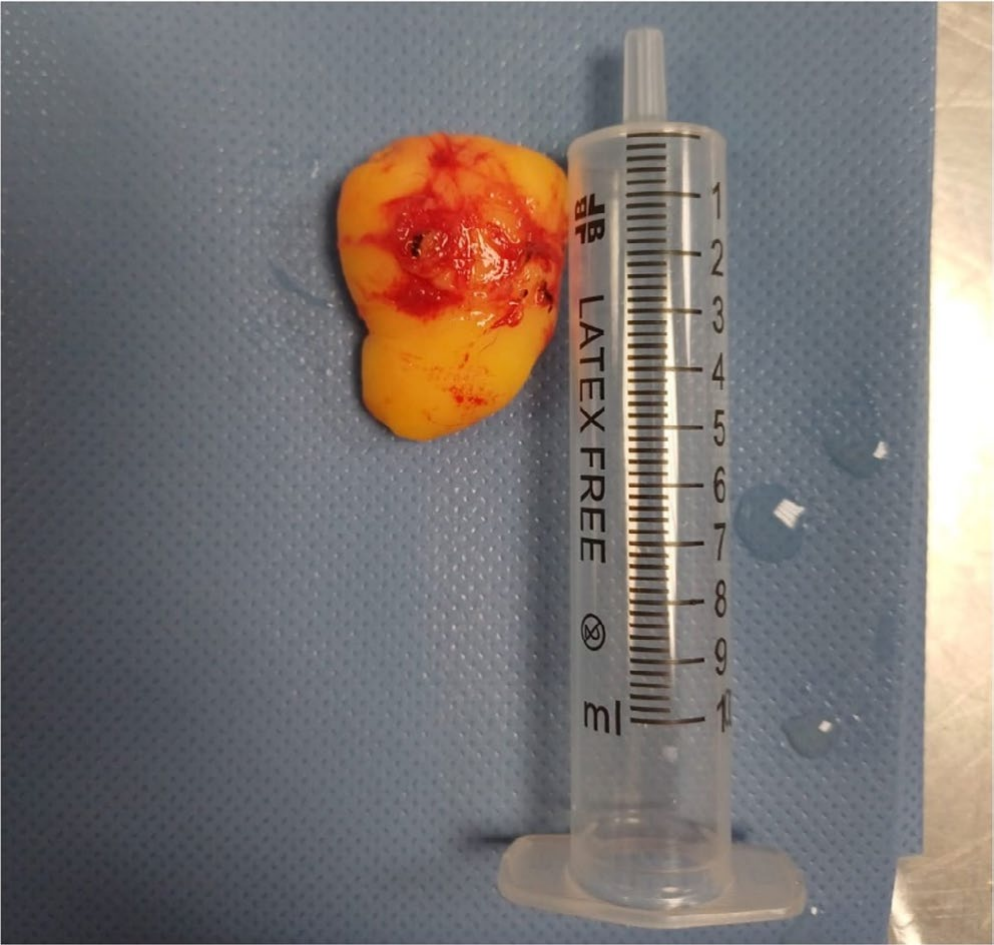
Gross appearance of the excised specimen showing a lobulated, yellow, encapsulated mass next to a 5‐mL syringe for size reference.

Macroscopically, it appeared as a well‐encapsulated, lobulated, yellowish mass, suggestive of adipose origin (Figure 3).

## 4 Conclusion and Results (Outcome and Follow‐Up)

**FIGURE 4:**
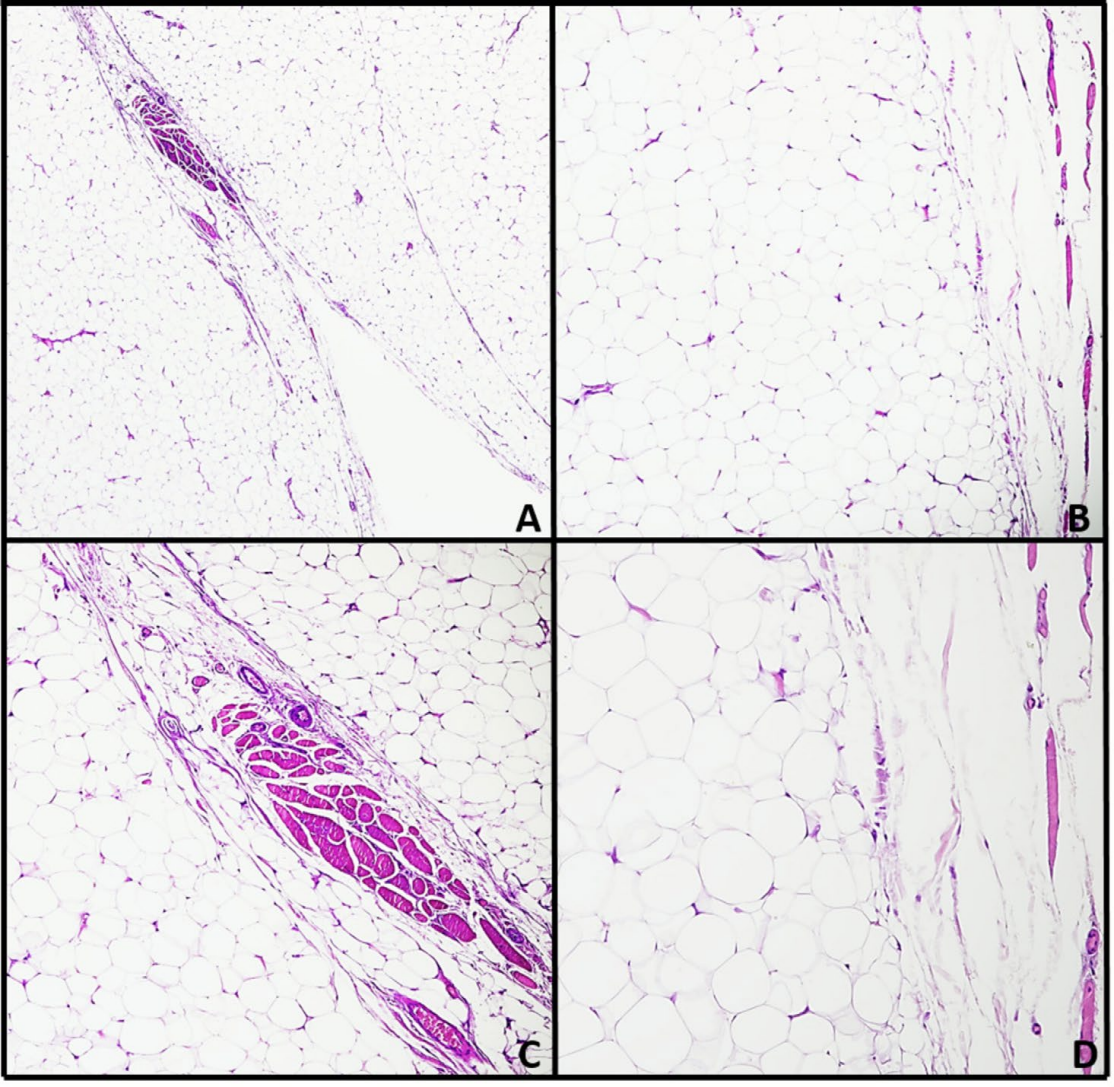
The tongue lesion consists of a well‐differentiated lipomatous proliferation. (A) At low magnification, a mass of mature adipose tissue is visible (H&E, 4×). (B, C) Variably sized fibrous septa and muscle bundles are interspersed within the lesion (H&E, 10×). (D) Higher magnification reveals mature adipocytes without evidence of cellular atypia (H&E, 20×).

The histopathological examination confirmed the diagnosis of a conventional lipoma, composed of mature adipocytes arranged in lobules, encapsulated by a thin fibrous capsule, and lacking atypia, mitotic activity, or infiltrative growth (Figure 4). The postoperative course was uneventful. At the 2‐month follow‐up, the surgical site had healed appropriately, with complete recovery of tongue mobility and resolution of all previously reported functional discomfort.

## 5 Discussion

Lingual lipomas are a rare clinical finding, accounting for approximately 0.3% of all tongue neoplasms, primarily due to the limited presence of adipose tissue in this anatomical region [5, 6]. These tumors usually present as slow‐growing, painless, submucosal lesions that are well‐circumscribed and soft to tense‐elastic in consistency. Their color often appears yellowish through the mucosa due to the adipose content. Most patients are asymptomatic unless the lesion grows large enough to impair mastication, speech, or deglutition, as occurred in our case [2].

Histologically, lipomas are composed of mature adipocytes arranged in lobules and surrounded by a thin fibrous capsule. Variants such as fibrolipoma, spindle cell lipoma, angiolipoma, and intramuscular (infiltrating) lipoma may be encountered, particularly in larger or deeper lesions [3, 7]. The intramuscular lipoma subtype is especially relevant in the tongue due to its potential to infiltrate muscle fibers, posing a diagnostic challenge by mimicking liposarcoma both clinically and radiographically. Hence, histopathological analysis remains essential for a definitive diagnosis, especially to differentiate benign lipomas from atypical lipomatous tumors or liposarcomas, as emphasized in the WHO Classification of Tumours of Soft Tissue and Bone [8]. The differential diagnosis of submucosal tongue masses includes not only benign lesions like granular cell tumors, neurofibromas, and vascular malformations, but also malignant neoplasms such as liposarcoma, although exceedingly rare in this site [6, 9]. Surgical excision is the treatment of choice and is typically curative when the lesion is completely removed. Recurrence is rare in well‐encapsulated lesions, particularly when excised entirely with clear margins, as seen in our patient's case [4, 5, 10].

This case further reinforces the importance of considering lingual lipoma in the differential diagnosis of any persistent, painless, submucosal tongue mass and highlights the excellent prognosis of surgical management for this rare benign neoplasm.

To provide a more comprehensive context for our case, we conducted a focused literature review of lingual lipomas published from 2021 to 2024. Only cases explicitly involving the tongue were included, excluding studies that grouped all intraoral locations. The selected reports are summarized in Table 1 [4, 7, 11, 12, 13, 14, 15, 16, 17, 18, 19].

**TABLE 1:** Summary of reported cases of lingual lipomas.

Author (Year)	Age/Sex	Location	Size	Duration	Symptoms	Functional impairment	Histologic type
Maglitto et al. (2023) [4]	67/M	Tip of tongue	5 cm	20 years	Dysphagia, dysarthria	Yes	Intramuscular lipoma
Gandhi et al. (2023) [11]	50/M	Dorsal tongue	6 × 4 cm	2 years	Speech and swallowing difficulties	Yes	Classical lipoma
Shinde et al. (2024) [12]	52/M	Sublingual	2 × 1 cm	6 months	Dysphagia, dysarthria	Yes	Classical lipoma
Mousa et al. (2023) [13]	n.s./F	Anterior tongue	n.s.	n.s.	Asymptomatic mass	No	Spindle cell lipoma
Alomari et al. (2024) [15]	1/M	Tongue borders	n.s.	Since birth	Irritability, feeding difficulties	Yes	Fibrolipoma
Salih et al. (2021) [7]	53/F	Anterior tongue	n.s.	n.s.	Painless mass	No	Fibrolipoma
Yang et al. (2024) [14]	4/F	Mid‐tongue	n.s.	n.s.	Painless swelling	No	Classical lipoma
Shathur et al. (2022) [16]	75/M	Tongue (n.s.)	Large (n.s.)	n.s.	Asymptomatic	No	Classical lipoma
Russo et al. (2023) [17]	31/F	Under‐tongue region	n.s.	2 months	Painful swelling	No	Lipoma with cartilage metaplasia
Mansoor et al. (2022) [18]	18/F	Dorsum of tongue	n.s.	8 years	Swelling	n.s.	Fibrolipoma
Guo et al. (2025) [19]	57/M	Left side of the tongue	3 × 1.5 cm	5 months	Painless mass	No	Spindle cell/pleomorphic lipoma

This synthesis reveals that lingual lipomas are rare benign tumors that most frequently manifest as slow‐growing, well‐demarcated submucosal masses. Although some lesions remain asymptomatic for years, others may cause significant functional impairments such as dysphagia, dysarthria, or masticatory discomfort, particularly when they reach considerable size or affect anatomically critical areas.

Lesion sizes in the reviewed cases ranged from 2 to over 8 cm, with durations spanning several months to over 20 years. Histologically, most tumors were classified as conventional lipomas, although fibrolipomas and spindle cell variants were also reported. In all cases, surgical excision was curative and no recurrence was observed during follow‐up. In the literature, follow‐up periods after surgical excision of lingual lipomas range from 6 months to 5 years, with no documented recurrences in most cases. Recurrence is considered rare and usually related to incomplete excision, particularly in the infiltrating subtype [11, 16].

Our case aligns with this clinical spectrum: a slowly progressive mass with increasing functional disturbance over time. The lesion's size, location, and symptomatology are consistent with the more severe end of the clinical range described in recent literature, underscoring the importance of early diagnosis and timely surgical intervention in similar presentations.

Awareness of this entity is essential for clinicians, as its benign clinical appearance may overlap with that of more aggressive pathologies, and early recognition enables timely curative management.

## 6 Conclusion

Lingual lipomas, although rare, should be considered in the differential diagnosis of persistent, painless tongue masses. Clinical findings may suggest the diagnosis, but histopathology is mandatory to exclude malignancy. Complete surgical excision remains the treatment of choice and generally ensures excellent functional recovery and prognosis.

## Author Contributions

**Gabriele Delia:** methodology, supervision, writing – original draft. **Fabiana Battaglia:** conceptualization, data curation, writing – original draft, writing – review and editing. **Maria Lentini:** resources, visualization. **Mariausilia Franchina:** resources. **Ludovica Pepe:** resources. **Francesco Stagno d'Alcontres:** supervision, writing – original draft.

## Funding

The authors have nothing to report.

## Consent

Written informed consent was obtained from the patient for publication of this case report and accompanying images.

## Conflicts of Interest

The authors declare no conflicts of interest.

## Data Availability

All data generated or analyzed during this study are included in this published article. No additional data are available.
